# Health state utilities associated with attributes of treatments for hepatitis C

**DOI:** 10.1007/s10198-014-0649-6

**Published:** 2014-12-07

**Authors:** Louis S. Matza, Sandhya J. Sapra, John F. Dillon, Anupama Kalsekar, Evan W. Davies, Mary K. Devine, Jessica B. Jordan, Amanda S. Landrian, David H. Feeny

**Affiliations:** 1 Outcomes Research, Evidera, 7101 Wisconsin Avenue, Suite 1400, Bethesda, MD 20814 USA; 2 Global Health Economics and Outcomes Research, Bristol-Myers Squibb, Princeton, NJ USA; 3 NHS Tayside and Medical Research Institute, University of Dundee, Dundee, UK; 4 Outcomes Research, Evidera, London, UK; 5 Department of Economics, McMaster University, Hamilton, ON Canada

**Keywords:** Utility, Cost-utility, Hepatitis C, Time trade-off, Treatment process utility, Time horizon

## Abstract

**Background:**

Cost-utility analyses are frequently conducted to compare treatments for hepatitis C, which are often associated with complex regimens and serious adverse events. Thus, the purpose of this study was to estimate the utility associated with treatment administration and adverse events of hepatitis C treatments.

**Design:**

Health states were drafted based on literature review and clinician interviews. General population participants in the UK valued the health states in time trade-off (TTO) interviews with 10- and 1-year time horizons. The 14 health states described hepatitis C with variations in treatment regimen and adverse events.

**Results:**

A total of 182 participants completed interviews (50 % female; mean age = 39.3 years). Utilities for health states describing treatment regimens without injections ranged from 0.80 (1 tablet) to 0.79 (7 tablets). Utilities for health states describing oral plus injectable regimens were 0.77 (7 tablets), 0.75 (12 tablets), and 0.71 (18 tablets). Addition of a weekly injection had a disutility of −0.02. A requirement to take medication with fatty food had a disutility of −0.04. Adverse events were associated with substantial disutilities: mild anemia, −0.12; severe anemia, −0.32; flu-like symptoms, −0.21; mild rash, −0.13; severe rash, −0.48; depression, −0.47. One-year TTO scores were similar to these 10-year values.

**Conclusions:**

Adverse events and greater treatment regimen complexity were associated with lower utility scores, suggesting a perceived decrease in quality of life beyond the impact of hepatitis C. The resulting utilities may be used in models estimating and comparing the value of treatments for hepatitis C.

**Electronic supplementary material:**

The online version of this article (doi:10.1007/s10198-014-0649-6) contains supplementary material, which is available to authorized users.

## Introduction

The hepatitis C virus is one of the most common blood-borne infections worldwide [1–3]. It is estimated that up to 85 % of individuals who are infected with the hepatitis C virus develop a chronic hepatitis C (CHC) infection [4–6], which typically persists for an individual’s lifetime if left untreated [3]. Although a majority of patients with CHC are asymptomatic in early phases of the disease [7, 8], the disease can gradually progress to serious symptomatic life-threatening liver conditions such as cirrhosis and liver cancer [9–12].

A range of pharmaceutical treatments is available for CHC, with the treatment goal of sustained virological response (SVR) defined as no detectable hepatitis C virus 12 weeks after discontinuing therapy [13]. Current treatments are administered typically for 24–48 weeks in complex combination treatment regimens involving injections plus a substantial number of daily tablets that may need to be administered at precise intervals throughout the day [14–16]. CHC treatments are also frequently associated with serious side effects, such as anemia, depression, flu-like symptoms, and skin problems [17–20]. The complex treatment regimens and adverse event profiles of available treatments likely lead to non-adherence to CHC treatment [21–24], which is likely to reduce treatment effectiveness. However, the landscape of treatment options for CHC is expanding with the recent development and approval of new treatment regimens that are more tolerable, simpler to administer than previously available regimens, and have shorter treatment duration [25–30].

As these new treatments for CHC are introduced, it is important to compare their cost-effectiveness to previously available treatments in order to demonstrate their value to clinicians, payers, and health technology assessment agencies. Cost-effectiveness analyses focusing on CHC treatments are often cost-utility models, which include the preferences of individuals for various health states and treatment-related outcomes [31–37]. In cost-utility models, treatment outcome is quantified in terms of utilities, which are scores representing the strength of preferences for health states, anchored on a scale with 1 representing full health and 0 representing dead [38, 39]. Although published utilities are available to represent various severity levels of hepatitis C and related complications [40–44], little is known about the utility impact of treatment processes and adverse events associated with treatments for CHC. Only one study was located that examined some of these attributes, and it focused specifically on injection frequency and the adverse event of flu-like symptoms [45]. While results of this previous study are useful, they do not provide insight into the utility associated with the substantial pill burden and wide range of serious adverse events often associated with CHC treatments.

Therefore, the purpose of the current study was to estimate the utility or disutility (i.e., reduction in utility score) of a broad range of treatment administration attributes and common adverse events associated with hepatitis C treatments. Given that these treatments vary widely in terms of treatment burden and adverse event profile, it may be important to identify utilities associated with these attributes so that they can be represented accurately in cost-utility models comparing treatments. These treatment attribute differences may be particularly important for models focusing on CHC because pharmaceutical treatments often have similar efficacy as indicated by similar SVR rates [46, 47]. Even when two treatment regimens have similar efficacy, the patient experience can vary substantially due to differences in treatment process and adverse events, and these differences should be captured in cost-utility models. To identify these utility values in the current study, respondents were asked to rate hypothetical health state descriptions (often called vignettes) in time trade-off (TTO) interviews. This common utility assessment method is well-suited for isolating the impact on utility of specific treatment attributes that are unlikely to be captured by generic preference-based instruments such as the EQ-5D [48] or Health Utilities Index [49].

Although utilities are most frequently used to quantify preferences for health outcomes, there is a growing body of research focused on “process utilities”. These studies have examined the utility impact of the treatment process itself in addition to the utilities associated with specific symptoms, medical conditions, or treatment outcome. For example, studies have found that utilities are influenced by treatment modalities including surgical versus nonsurgical management [29]; inhaled versus injected treatment [50]; oral versus injectable treatment [51, 52]; dose frequency [51, 52]; inpatient versus outpatient treatment [53]; two types of prenatal genetic testing [54]; early-stage cervical cancer treatment options [55]; and specific medication options [56]. These studies have been able to detect differences in preference, and more convenient treatment processes are generally associated with greater utility values. The current study adds to this developing literature on process utilities.

## Methods

### Study overview

Chronic hepatitis C (CHC) health state descriptions were drafted based on literature review and input from clinicians. Then, the health states were refined based on additional clinician interviews and a pilot study conducted with general population respondents in London, UK. Finally, health states were rated in a TTO valuation study with general population participants in Edinburgh and London, UK. All participants completed the TTO valuations twice, including once with a 10-year time horizon and once with a 1-year time horizon. A 10-year time horizon was used to maximize comparability with previously published utility studies. Participants also rated the health states in a TTO task with a 1-year time horizon in order to obtain preferences for health states lasting for a period of time which more closely mirrors typical treatment duration.

### Health state development

Health state descriptions were drafted based on interviews with clinicians and literature review. Telephone interviews were conducted with three clinicians who specialized in treatment of patients with hepatitis C. Two of the clinicians were from the US, while the other was based in the UK. Interviews were first conducted with all three clinicians to inform health state development, with questions focusing on patients’ typical experiences with hepatitis C, the range of available treatment regimens, and common treatment-related adverse events. After health states were drafted based on clinician input, the draft health states were sent to the three clinicians so that they could review the text and comment on its clarity and accuracy. Minor revisions were made based on these comments.

Literature review was conducted throughout the health state development process to inform the clinician interview questions and ensure that the health state descriptions were consistent with published research. Literature searches focused on the symptoms and impact of hepatitis C [4, 8, 10]; treatment regimens of available and experimental pharmaceutical treatments for hepatitis C [7, 15, 25]; and treatment-related adverse events that clinicians considered to be most common and bothersome for patients [17, 18, 20, 57–60]. Literature was initially identified by searching for relevant terms in MEDLINE, and additional articles were suggested by the three clinicians who were interviewed for this study.

Health states were tested in a pilot study conducted with 24 general population participants in London, UK (9 female; mean age = 26.3 years; age range = 19–45 years) recruited via newspaper and online advertisements. Each participant valued the states using multiple utility assessment methods, including TTO with two time horizons (1- and 10-year), standard gamble (SG), chaining approaches (i.e., TTO and SG using an undesirable living health state rather than dead as a lower anchor), and a path state approach (i.e., a sequence of health states grouped into a single life span rated with TTO). The order in which participants completed tasks was randomized (11 completed SG first; 8 completed TTO first). Most methods yielded utility scores in a reasonable range with logical discrimination among health states. Based on these results, the TTO method was selected for use in the subsequent main study with a larger sample because it was relatively easy for participants to understand and complete, and because it is consistent with the methods used in many recent utility valuation studies, including the influential measurement and valuation of health (MVH) study that identified utilities of EQ-5D health states [61, 62]. Participants consistently reported that the health states were clear and easy to understand. Some participants suggested minor revisions in formatting and word choice, and the health states were edited accordingly.

Results of the pilot study were presented to clinicians, who were asked if they believed the differences among health state utilities were a reasonable representation of patients’ experiences. All clinicians agreed that the pilot study results were logical. For example, the adverse event of depression was associated with a relatively large disutility, and clinicians thought this was justified given the severity level of depression that can emerge as a side effect of CHC treatment.

### Final health states administered in TTO interviews

A total of 14 health states were administered (see Electronic Supplement Appendix A for full health state text). All health states included the same description of hepatitis C, including explanation of the virus, long-term risks, indication that the condition is currently asymptomatic, and a brief description of fear and stress that can accompany the disease. The first four health states, labeled A–D, included additional statements briefly describing oral-only treatment regimens ranging from one tablet per day (A) to seven tablets per day (D). These oral-only health states were designed to be consistent with regimens of recently tested and/or approved treatments [25, 27, 63].

The next four health states, E–H, described treatment regimens including oral and injectable medication. These health states were designed to represent the most common treatment regimens, which include varying numbers of orally administered tablets plus weekly interferon injections [18, 64]. Numbers of tablets in these health states ranged from 7 to 18 per day. The health states with a 12-tablet daily regimen (F and G) were designed to represent telaprevir plus ribavirin [65, 66], while the 18-tablet daily regimen (H) corresponds to treatment with boceprivir plus ribavirin [67–69]. Because the requirement to take telaprevir with fatty food may be aversive to some patients [16, 65, 66], the telaprevir treatment regimen was presented with (F) and without (E) this treatment attribute so that the disutility of the fatty food requirement could be calculated.

The final six health states (I–N) described a treatment regimen identical to health state E (seven tablets daily plus weekly injections), but each of these six health states added a single adverse event. Two of these health states described mild (I) and severe (J) anemia, which is an adverse event associated with several common CHC treatments, including ribavirin [17], alpha interferon [17], and protease inhibitors such as telaprevir [57, 70] and boceprevir [70]. Health state K described flu-like symptoms emerging after weekly injections, which is common with alpha interferon treatment [18, 71, 72]. Health states L and M described mild and severe rashes, which have been shown to be related to treatment with ribavirin [18, 73] and telaprevir [58, 65]. The rash that emerges with telaprevir can be particularly severe, as represented in health state M [59, 65]. Finally, health state N described depression, which has previously been reported in patients treated with alpha interferon [19] and ribavirin [18, 60].

### Participants

Participants were required to be (1) at least 18 years old; (2) able to understand the assessment procedures; (3) able and willing to give written informed consent; and (4) residing in the UK. Inclusion criteria did not specify particular clinical characteristics because interviews were intended to yield utilities that may be used in cost-utility analyses for submission to health technology assessment agencies such as the National Institute for Health and Care Excellence (NICE), most of whom prefer that utilities represent general population values [74–77].

Participants were recruited via advertisements in three newspapers in Edinburgh, two newspapers in London, and the website http://www.gumtree.com/. A total of 585 individuals responded to the advertisements, and 252 of these were reached for screening. Of the 252 screened participants, 251 were eligible, 215 were scheduled for interviews, and 188 participants attended interviews. Of the 188 participants, 6 were unable to complete the TTO interview procedures. Thus, a total of 182 valid interviews were completed.

### Utility interview procedures and scoring

Utilities were derived by eliciting values for the health state descriptions in a TTO utility interview. In TTO procedures, the duration of time spent in the health state being rated (i.e., the time horizon) is an important component of the task. This time horizon varies across TTO studies. The most commonly used TTO time horizon appears to be 10 years, which is likely favored because of its simplicity for interviewers and respondents as well as because it was used in the MVH study that elicited utilities for EQ-5D health states [62, 78]. However, other time horizons are also frequently used, ranging from 1 or 2 years to longer time horizons based on each respondent’s life expectancy [79–85].

In the current study, TTO interviews were completed with two time horizons. All participants rated the complete set of health states twice, with a 10-year time horizon and a 1-year time horizon. The 10-year time horizon was used to maximize comparability with previously published TTO utility studies, including the MVH study [62, 78]. However, all health states included a description of a hepatitis C treatment course, which typically lasts approximately 24–48 weeks [16]. Therefore, the shorter time horizon was used so that health states would be rated in a TTO task that more closely matches the true clinical timeframe of the health states. To control for order effects, participants were randomly assigned to complete either the 10- or 1-year TTO first, followed by the other time horizon.

To introduce participants to the health state descriptions, a ranking exercise was conducted. After each participant ranked the health states in order of preference, health state utilities were obtained using the TTO method. Health states were not presented with the organized lettering system used in current tables (i.e., A–N). Instead, health states were numbered in a random order so that lettering/numbering would not provide an indication of which health states might be more or less preferable.

For TTO ratings of each health state, participants were offered a choice between spending a 10- or 1-year period in the health state versus spending shorter amounts of time in the full health state (1-year increments in the 10-year TTO; 1-month increments in the 1-year TTO). For each health state, choices were presented in an order that alternated between longer and shorter durations in full health (e.g., 10, 0, 9, 1, 8, 2, 7, 3, 4, and 5 years). Each health state rated as better than dead received a utility value on a scale with the anchors of dead (0) and full health (1). The assigned value was calculated based on the choice in which the respondent is indifferent between *y* years/months in the health state being evaluated and *x* years/months in full health (followed by dead). The resulting utility estimate (*u*) is calculated as *u* = *x*/*y*.

If participants indicated that a health state was worse than dead, the interviewer altered the task so that respondents were offered a choice between immediate death (alternative 1) and a 10-year/1-year life span (alternative 2) beginning with varying amounts of time in the health state being rated, followed by full health for the remainder of the time horizon. For these health states, the current study used a bounded scoring approach, which is commonly used to avoid highly skewed distributions for negative utilities [86]. This scoring approach limits the utility range of health states worse than dead to values between 0 and −1. To compute these bounded negative utility values, the current study used the Dolan method [78] as described by Rowen and Brazier [39]. This method uses the formula *u* = −*x*/*t*, where *x* is the period of time in full health, and* t* is the total life span of alternative 2 in the TTO choice. In the current study, *t* was 10 and 1 year, which was the period of time in the health state being rated plus subsequent years/months in full health.

### Data collection and statistical analysis procedures

Interviews were conducted in private conference rooms in London and Edinburgh in June 2013. All procedures and materials were approved by an independent Institutional Review Board, and every participant provided written informed consent before completing any study procedures. All interviews were conducted by the project manager or other trained members of the project team. The interviews followed a standardized interview guide, and the TTO choices were presented with the use of booklets in which each page had an image depicting a different TTO choice. Participants completed a brief demographic and clinical form, followed by the TTO utility interview described above. Statistical analyses were completed using SAS version 9.2 (SAS Institute, Cary, NC).

Continuous variables, including utilities and pairwise differences between health state utilities, are summarized in terms of means and standard deviations, and categorical variables such as gender and racial/ethnic background are summarized as frequencies and percentages. Demographic characteristics of the London and Edinburgh subgroups were compared with Chi-square analyses (for categorical variables) and *t* tests (for continuous variables).

Utility differences were examined for pairs of health states that were directly comparable to each other. The difference between two health states that differ in only one treatment attribute represents the disutility (i.e., decrease in utility score) or added utility (i.e., increase in utility score) associated with that treatment attribute. For example, health states D and E are identical except for the addition of a weekly injection to health state E. Therefore, the difference between health states D and E represents the disutility of a weekly injection in the context of treatment for hepatitis C. In addition, pairwise comparisons between health states were conducted using *t* tests to examine whether utility differences were statistically significant.

## Results

### Sample description

The total sample included 182 participants with a mean age of 39.3 years (SD = 15.1) (Table 1). The sample was evenly split between men (*n* = 91) and women (*n* = 91). The majority of participants reported ethnicity as white (74.7 %), and more participants reported being single (56.0 %) than married (28.0 %). Most participants reported being employed (26.9 % full-time and 26.9 % part-time). Half of the sample had completed a university degree (*n* = 91; 50.0 %). When asked to report health conditions, the most common responses were depression (*n* = 15; 8.2 %), anxiety (*n* = 8; 4.4 %), arthritis (*n* = 8; 4.4 %), hypertension (*n* = 7; 3.8 %), and cancer (*n* = 7; 3.8 %). One respondent reported having hepatitis C (0.05 %) and another reported having hepatitis B (0.05 %).

**Table 1:** Sample demographic characteristics

Demographic characteristics	Edinburgh subgroup (*N* = 88)	London subgroup (*N* = 94)	Total sample (*N* = 182)	*P* value*
Age (mean, SD)	41.0, 14.9	37.8, 15.3	39.3, 15.1	0.15
Gender (*n*, %)				
Female	47 (53.4 %)	44 (46.8 %)	91 (50.0 %)	0.37
Male	41 (46.6 %)	50 (53.2 %)	91 (50.0 %)	
Ethnicity (*n*,%)				
White	83 (94.3 %)	53 (56.4 %)	136 (74.7 %)	<0.0001
Mixed	2 (2.3 %)	9 (9.6 %)	11 (6.0 %)	
Asian	3 (3.4 %)	11 (11.7 %)	14 (7.7 %)	
Black	0	18 (19.1 %)	18 (9.9 %)	
Other	0	3 (3.2 %)	3 (1.6 %)	
Marital status (*n*, %)				
Single	46 (52.3 %)	56 (59.6 %)	102 (56.0 %)	0.20
Married	30 (34.1 %)	21 (22.3 %)	51 (28.0 %)	
Other	12 (13.6 %)	17 (18.1 %)	29 (15.9 %)	
Employment status (*n*, %)				
Full-time work	24 (27.3 %)	25 (26.6 %)	49 (26.9 %)	0.97
Part-time work	23 (26.1 %)	26 (27.7 %)	49 (26.9 %)	
Other	41 (46.6 %)	43 (45.7 %)	84 (46.2 %)	
Education level (*n*, %)				
No formal qualifications	4 (4.5 %)	1 (1.1 %)	5 (2.7 %)	0.046
GCSE/O-levels or equivalent	11 (12.5 %)	11 (11.7 %)	22 (12.1 %)	
A-levels or equivalent	10 (11.4 %)	27 (28.7 %)	37 (20.3 %)	
Vocational/work-based qualifications	6 (6.8 %)	9 (9.6 %)	15 (8.2 %)	
University degree	29 (33.0 %)	29 (30.9 %)	58 (31.9 %)	
Post-graduate degree (MA, PhD, PGCE)	21 (23.9 %)	12 (12.8 %)	33 (18.1 %)	

* *P* values are based on *t* tests for continuous variables and Chi-square analyses for categorical variables, comparing the London and Edinburgh subgroups

There were no significant differences between the London (*n* = 94) and Edinburgh (*n* = 88) samples in age, gender, marital status, or employment status. However, the Edinburgh sample had a significantly higher percentage of white participants than the London sample (94.3 vs 56.4 %; *P* < 0.001). There was also a significant difference in education level, as the Edinburgh subgroup was more likely to have attained a university or post-graduate degree (*P* = 0.046).

### Health state ranking

In the introductory ranking task, rankings ranged from 1 (most preferable health state) to 14 (least preferable health state). On average, greater treatment regimen complexity was associated with lower rankings, and health states with adverse events were ranked below health states without adverse events. Mean rankings for each of the 14 health states were as follows (in order of most preferable to least preferable): health state A (mean ranking = 1.02); B (1.99); C (2.99); D (4.01); E (5.00); F (6.27); G (7.68); H (8.23); I (9.40); L (9.85); K (10.76); J (12.02); N (12.78); M (13.01).

### Health state utilities (10-year time horizon)

Health state utility scores are presented in Table 2. All 182 participants had complete utility data for the full set of 14 health states. The health states describing all-oral treatment regimens had the highest 10-year TTO utility values, ranging from 0.79 for seven tablets per day (health state D) to 0.80 for one tablet per day (A). Health states with weekly injections had lower utility values than health states with all-oral regimens, ranging from 0.71 (H: weekly injection + 18 tablets per day) to 0.77 (E: weekly injection + seven tablets per day).

**Table 2:** Time trade-off (TTO) health state utilities (*N* = 182)

Hepatitis C health states	TTO with 10-year time horizon		TTO with 1-year time horizon	
	Mean	SD	Mean	SD
Health states differing by treatment regimen				
A. All-oral regimen (1 tablet per day)	0.80	0.30	0.81	0.29
B. All-oral regimen (2 tablets per day)	0.80	0.30	0.81	0.29
C. All-oral regimen (3 tablets per day)	0.79	0.30	0.80	0.29
D. All-oral regimen (7 tablets per day)	0.79	0.30	0.79	0.29
E. Oral treatment (7 tablets per day) + weekly injection	0.77	0.30	0.77	0.30
F. Oral treatment (12 tablets per day) + weekly injection	0.75	0.31	0.76	0.32
G. Oral treatment (12 tablets per day taken with fatty food) + weekly injection	0.71	0.35	0.72	0.35
H. Oral treatment (18 tablets per day) + weekly injection	0.71	0.35	0.72	0.33
Health states differing by adverse events				
I. Health state E + mild anemia	0.65	0.36	0.65	0.38
J. Health state E + severe anemia	0.45	0.42	0.47	0.41
K. Health state E + flu-like symptoms	0.56	0.39	0.57	0.39
L. Health state E + mild rash	0.65	0.40	0.66	0.37
M. Health state E + severe rash	0.30	0.50	0.34	0.47
N. Health state E + depression	0.31	0.48	0.33	0.48

TTO scores are on a scale anchored with 0 representing dead and 1 representing full health

All health states with adverse events (I–N) had lower 10-year TTO utility values than any of the health states without adverse events (A–H). Among the adverse event health states, the highest utility scores were for I (mild anemia) and L (mild rash), which both had mean 10-year utilities of 0.65. The lowest utility values were for health states describing depression (health state N; utility = 0.31) and severe rash (M; 0.30).

### Health state utilities (1-year time horizon)

The mean 1-year TTO utility scores were similar to the 10-year scores, or in some cases, identical when rounded to two decimal places (Table 2). For 13 of the 14 health states, the 1- and 10-year scores had a utility difference of 0.02 or less. The only health state with a larger difference between the time horizons was health state M (severe rash), with a difference of 0.04 between the 10- and 1-year scores.

### Comparisons between pairs of health states differing in treatment administration attributes

Difference scores were computed to identify the disutility associated with various treatment attributes. All pairwise difference scores between health states varying in treatment administration attributes (health states A–H) are presented in Table 3. In addition, differences between pairs of health states that vary in only one treatment attribute are examined with *t* tests in Table 4. Because the health state pairs in Table 4 were identical except for a single difference in treatment regimen, any difference in utility score represents the impact of the treatment attribute differences on respondent preference. The 10- and 1-year analyses followed similar patterns, and both sets of results are presented in Tables 3 and 4. The 10-year results are summarized here.

**Table 3:**
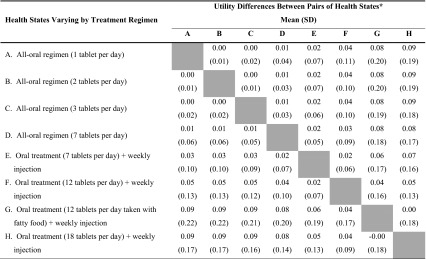
Utility differences between pairs of health states varying by treatment regimen (*N* = 182)

* Difference scores above the gray shaded cells were computed with 10-year TTO utility scores. Difference scores below the gray shaded cells were computed with 1-year TTO utility scores. Difference scores were computed by subtracting health states with letters closer to the end of the alphabet from health states with letters closer to the beginning of the alphabet (e.g., A–B, A–C, B–C)

**Table 4:** *T* tests comparing pairs of health states differing in treatment administration attributes (*N* = 182)

Comparison	Hepatitis C health states	TTO with 10-year time horizon			TTO with 1-year time horizon		
		Mean utility^a^	Mean (SD) difference score	*P* value^b^	Mean utility^a^	Mean (SD) difference score	*P* value^b^
A vs B	A. 1 tablet per day	0.80	0.00 (0.01)	0.32	0.81	0.00 (0.01)	0.32
	B. 2 tablets per day	0.80			0.81		
A vs C	A. 1 tablet per day	0.80	0.00 (0.02)	0.059	0.81	0.00 (0.02)	0.041
	C. 3 tablets per day	0.79			0.80		
A vs D	A. 1 tablet per day	0.80	0.01 (0.04)	0.0036	0.81	0.01 (0.06)	0.0032
	D. 7 tablets per day	0.79			0.79		
B vs C	B. 2 tablets per day	0.80	0.00 (0.01)	0.045	0.81	0.00 (0.02)	0.071
	C. 3 tablets per day	0.79			0.80		
B vs D	B. 2 tablets per day	0.80	0.01 (0.03)	0.0026	0.81	0.01 (0.06)	0.0043
	D. 7 tablets per day	0.79			0.79		
C vs D	C. 3 tablets per day	0.79	0.01 (0.03)	0.0036	0.80	0.01 (0.05)	0.0068
	D. 7 tablets per day	0.79			0.79		
D vs E	D. 7 tablets per day	0.79	0.02 (0.05)	<0.0001	0.79	0.02 (0.07)	0.0002
	E. 7 tablets per day + weekly injection	0.77			0.77		
E vs F	E. 7 tablets per day + weekly injection	0.77	0.02 (0.06)	0.0002	0.77	0.02 (0.07)	0.0005
	F. 12 tablets per day + weekly injection	0.75			0.76		
E vs H	E. 7 tablets per day + weekly injection	0.77	0.07 (0.16)	<0.0001	0.77	0.05 (0.13)	<0.0001
	H. 18 tablets per day + weekly injection	0.71			0.72		
F vs H	F. 12 tablets per day + weekly injection	0.75	0.05 (0.13)	<0.0001	0.76	0.04 (0.09)	<0.0001
	H. 18 tablets per day + weekly injection	0.71			0.72		
F vs G	F. 12 tablets per day + weekly injection	0.75	0.04 (0.16)	0.0005	0.76	0.04 (0.17)	0.0024
	G. 12 tablets per day (taken with fatty food) + weekly injection	0.71			0.72		

^a^TTO scores are on a scale anchored with 0 representing dead and 1 representing full health

^b^ *P* values are based on *t* tests comparing two utility means

In general, health states describing more complex and burdensome treatment regimens were associated with lower utility values. While some treatment regimen differences had virtually no impact on utility (e.g., one tablet vs. three tablets per day), other treatment regimen differences were associated with substantial utility differences. For example, the difference between the least burdensome regimens (health states A, B, and C) and most burdensome regimens (health states G and H) was 0.09 (Table 3).

Among the all-oral treatment regimen health states (A through D), the magnitude of utility differences was minimal (i.e., rounding to either 0.01 or 0.00) despite reaching statistical significance in some cases. The addition of a weekly injection to an otherwise identical health state (i.e., comparison between D and E) resulted in a statistically significant utility difference of 0.02 (*P* < 0.001). Among regimens including weekly injections, differences in the number of tablets were associated with statistically significant utility differences (health states E vs. F, E vs. H, and F vs. H; all *P* < 0.001). Adding the fatty food requirement to a 12-tablet daily regimen resulted in a statistically significant utility reduction (difference = 0.04; *P* < 0.001) (Table 4).

### Comparisons between pairs of health states differing in adverse events

The disutility of each adverse event was computed by subtracting the utility of each adverse event health state (I through N) from the utility of health state E (Table 5). The adverse event health states were identical to health state E other than the addition of the adverse event. Thus, the utility difference between health state E and these other health states represents the impact of each adverse event on respondent preference. The 10- and 1-year analyses followed similar patterns (Table 5), and the 10-year results are summarized here.

**Table 5:** *T* tests comparing pairs of health states differing in adverse events (*N* = 182)

Comparison	Hepatitis C health states	TTO with 10-year time horizon			TTO with 1-year time horizon		
		Mean utility^a^	Mean (SD) difference score	*P* value^b^	Mean utility^a^	Mean (SD) difference score	*P* value^b^
E vs I	E. 7 tablets per day plus injectable medication	0.77	0.12 (0.21)	<0.0001	0.77	0.12 (0.23)	<0.0001
	I. health state E + mild anemia	0.65			0.65		
E vs J	E. 7 tablets per day plus injectable medication	0.77	0.32 (0.33)	<0.0001	0.77	0.30 (0.31)	<0.0001
	J. health state E + severe anemia	0.45			0.47		
E vs K	E. 7 tablets per day plus injectable medication	0.77	0.21 (0.27)	<0.0001	0.77	0.20 (0.26)	<0.0001
	K. health state E + flu-like symptoms	0.56			0.57		
E vs L	E. 7 tablets per day plus injectable medication	0.77	0.13 (0.26)	<0.0001	0.77	0.12 (0.21)	<0.0001
	L. health state E + mild rash	0.65			0.66		
E vs M	E. 7 tablets per day plus injectable medication	0.77	0.48 (0.44)	<0.0001	0.77	0.43 (0.41)	<0.0001
	M. health state E + severe rash	0.30			0.34		
E vs N	E. 7 tablets per day plus injectable medication	0.77	0.47 (0.42)	<0.0001	0.77	0.45 (0.42)	<0.0001
	N. health state E + depression	0.31			0.33		
L vs M	L. health state E + mild rash	0.65	0.35 (0.38)	<0.0001	0.66	0.32 (0.37)	<0.0001
	M. health state E + severe rash	0.30			0.34		
I vs J	I. health state E + mild anemia	0.65	0.20 (0.26)	<0.0001	0.65	0.18 (0.22)	<0.0001
	J. health state E + severe anemia	0.45			0.47		

^a^TTO scores are on a scale anchored with 0 representing dead and 1 representing full health

^b^ *P* values are based on *t* tests comparing two utility means

All adverse event health state utilities were significantly different from the utility of health state E (all *P* < 0.001). The smallest disutilities were for mild anemia (health state I; difference score = 0.12) and mild rash (L; 0.13), while the greatest disutilities were for depression (N; 0.47) and severe rash (M; 0.48).

Two types of adverse events, rash and anemia, were presented in separate health states describing mild and severe conditions. In both cases, the severe adverse event had a significantly lower utility value than the mild adverse event (*P* < 0.001), with difference scores of 0.35 between mild and severe rash and 0.20 between mild and severe anemia.

## Discussion

Both the 10- and 1-year TTO methods were able to detect differences in health state preference associated with treatment regimens and adverse events. More complex and burdensome treatment regimens, including increased pill burden, addition of injectable treatment, and a fatty food requirement, were associated with lower utilities. This finding adds to the growing body of literature on *process utilities*, which quantify the impact of treatment process characteristics such as mode of administration and dose frequency [87, 88]. While treatment process is likely to have less impact on utility than efficacy or safety, the relatively small utility differences associated with treatment process can influence the outcome of a cost-utility analysis, particularly when modeling large numbers of patients.

While treatment regimen differences were associated with utility differences up to 0.09, adverse event disutilities were larger, ranging from 0.12 to 0.48. Although some of these disutility values may initially seem larger than expected, their magnitude is reasonable given the severity of these adverse events. For example, health state N describing moderate to severe depression had a utility value of 0.31, resulting in a disutility of 0.47. Previous studies of depression utility outside the context of CHC have found similarly low utility estimates for moderate and severe depression [89–92]. In the current study, the lowest rated health state had a mean utility of 0.30, which seems reasonable given that the health state describes a very severe rash that can occur as a side effect of treatment with telaprevir [58, 59, 65].

Studies identifying minimally important differences (MID) in utility have focused on utilities derived from generic preference-based measures such as the EQ-5D, HUI, and SF-6D. For these instruments, MIDs have generally been reported in a range from 0.01 to 0.08 [93–97]. Less formal estimates of clinically important differences in direct utility elicitation (e.g., TTO methods) have suggested thresholds of 0.05–0.10 [38, 98]. Clearly, the disutility of every adverse event examined in the current study exceeds criteria for an important utility difference, indicating that it is important to quantify the impact of these side effects in cost-utility models. Some of the utility differences among treatment regimens also exceed MID estimates for utilities (Table 3).

Because all health states were rated in TTO tasks with 10- and 1-year time horizons, this study offers a unique opportunity to compare between two time horizons for multiple health states. Several previous studies have found that the TTO time horizon can influence results, and different time horizons may lead to different utility scores [61, 99–103]. In the current study, one might expect that the 1- and 10-year time horizons could generate different preference scores. For example, some symptoms, adverse events, or treatment regimens may seem more tolerable for shorter durations than for longer periods of time. However, in contrast to previous literature suggesting that different time horizons may yield different results, the current study found that 10- and 1-year utility scores were remarkably similar to each other (Table 2). The consistent results across time horizons suggest that the health state utilities were robust and stable, regardless of the time horizon used in the TTO task. Because the 1-year time horizon more closely corresponds to a course of hepatitis C treatment than the 10-year timeframe, researchers may prefer to use the 1-year values in the base case analysis of a cost-utility model.

Despite logical results, some study design characteristics suggest that findings should be interpreted with appropriate caution. The hypothetical health state approach is limited by the accuracy and level of detail in the health state descriptions. In addition, this approach has a potential focusing effect, which could lead respondents to attend more closely to small differences among health states. Still, this method was used because it is well-suited for isolating utility impact of specific treatment-related attributes, in contrast to generic measures such as the EQ-5D, which may not be sensitive to these attributes. However, the extent to which current utilities would correspond to patients’ ratings of their own health is not known. If comparability between current utilities and EQ-5D derived utilities is important for a particular cost-utility model, it is recommended that modelers use the utility values derived in the 10-year TTO assessment, which is consistent with the methods originally used to value EQ-5D health states [78].

Another possible limitation is that all health states explicitly named the disease that was described (i.e., “diagnosed with hepatitis C”). Some studies have suggested that including the disease label in a health state can influence utility scores, although other studies have reported situations when the label did not affect valuations [104–106]. To avoid risk of influencing utility values, some researchers recommend omitting the disease label from health states, while others include the label to make the health state as clear as possible. One advantage of including the label is that health states with the disease name more closely represent the patient experience because patients typically know the name of their condition. In the current study, it was determined that the health states should include the label because most patients with hepatitis C are asymptomatic. Therefore, the label was necessary to emphasize that each health state described a patient with a serious medical condition, despite a lack of symptoms.

Two other health state design decisions were also made because hepatitis C is usually asymptomatic. Whenever possible, health states should avoid statements involving uncertainty, which could increase variance and error because respondents’ interpretations of the uncertain statements could vary. In addition, it is usually best for health states to focus on description, without telling the respondent how to feel about the medical condition, in order to avoid biasing the responses. However, because hepatitis C is asymptomatic in most cases, health states for the current study included long-term risks (e.g., liver damage) and emotional impact (e.g., fear of future risks) in order to underscore the potential seriousness of this condition despite the lack of symptoms. While including risks and emotional impact could lead to error or biased health state valuations, these statements were identical in all 14 health states. Therefore, these health state characteristics would not have biased key results, which are the differences between health state pairs rather than the absolute value of any individual health state utility.

Two additional limitations of the adverse event health states should be noted. First, adverse events were all added to the treatment regimen described in health state E (i.e., seven daily tablets plus weekly injection). It is possible that the adverse event disutilities could be different if the adverse events were added to health states with different treatment regimens. However, in the current study, it was not feasible to add additional health states to an interview that was already quite complex with 14 health states and two time horizons. Second, although patients treated for hepatitis C may experience multiple adverse events, disutility estimates in the current study represent the utility decrease associated with a single adverse event. Current results do not provide insight into the utility impact of multiple simultaneous adverse events.

Despite these limitations, the current study provides utility scores that may be used in cost-utility modeling to provide a more detailed representation of experience with hepatitis C treatment. Both 10- and 1-year TTO methodology yielded health state utilities in a reasonable range with logical discrimination between health states. By using the current utility scores, models can quantify the impact of treatment regimen and adverse events when comparing the value of new and previously available treatments.

## Electronic supplementary material

Below is the link to the electronic supplementary material.

Supplementary material 1 (DOCX 26 kb)
