# Supplementary material for: Health state utilities associated with attributes of treatments for hepatitis C
Source: Eur J Health Econ. 2014 Dec 7;16(9):1005–18. doi: 10.1007/s10198-014-0649-6 (PMC4646927; doi:10.1007/s10198-014-0649-6)
Supplement: Supplementary file 1 — Supplementary material 1 (DOCX 26 kb) [file 10198_2014_649_MOESM1_ESM.docx]

**APPENDIX A: FULL TEXT OF ALL HEALTH STATES**

**HEALTH STATE A:**

**Hepatitis C**

- You have been diagnosed with hepatitis C.
- Hepatitis C is a virus that gets into your blood and attacks your liver cells. If it is not treated, it can cause liver damage and eventually lead to cancer. It could also cause you to need a liver transplant.
- You do not yet notice symptoms of your hepatitis C.

**Impact**

- You live with fear of the future because of the problems that could be caused by Hepatitis C.
- Hepatitis C is an infectious disease that can be passed to others through sexual contact. This causes you stress.

**Treatment**

- In the hope of avoiding serious problems, you are being treated for hepatitis C. You are treated with oral medication.
- You take 1 tablet per day.

**Side Effects**

- You do not notice any side effects of the medication.

**HEALTH STATE B:**

Same hepatitis C and impact statements as Health State A.

**Treatment**

- In the hope of avoiding serious problems, you are being treated for hepatitis C. You are treated with oral medication.
- You take 2 tablets per day: 1 in the morning, and 1 in the evening.

**Side Effects**

- You do not notice any side effects of the medication.

**HEALTH STATE C:**

Same hepatitis C and impact statements as Health State A.

**Treatment**

- In the hope of avoiding serious problems, you are being treated for hepatitis C. You are treated with oral medication.
- You take 3 tablets per day: 1 in the morning, and 2 in the evening.

**Side Effects**

- You do not notice any side effects of the medication.

**HEALTH STATE D:**

Same hepatitis C and impact statements as Health State A.

**Treatment**

- In the hope of avoiding serious problems, you are being treated for hepatitis C. You are treated with oral medication.
- You take 7 tablets per day: some in the morning, some in the evening.

**Side Effects**

- You do not notice any side effects of the medication.

**HEALTH STATE E:**

Same hepatitis C and impact statements as Health State A.

**Treatment**

- In the hope of avoiding serious problems, you are being treated for hepatitis C. You are treated with oral and injectable medication.
- You take 7 tablets per day: some in the morning, some in the evening.
- You also receive an injection once each week. This can occur in your doctor’s office, or you can give yourself the injection at home.

**Side Effects**

- You do not notice any side effects of either medication.

**HEALTH STATE F:**

Same hepatitis C and impact statements as Health State A.

**Treatment**

- In the hope of avoiding serious problems, you are being treated for hepatitis C. You are treated with oral and injectable medication.
- You take 12 tablets per day: some in the morning, some in the evening.
- You also receive an injection once each week. This can occur in your doctor’s office, or you can give yourself the injection at home.

**Side Effects**

- You do not notice any side effects of either medication.

**HEALTH STATE G:**

Same hepatitis C and impact statements as Health State A.

**Treatment**

- In the hope of avoiding serious problems, you are being treated for hepatitis C. You are treated with oral and injectable medication.
- You take 12 tablets per day: some in the morning, some in the evening.
- The tablets must always be taken with fatty foods.
  - For example, you will need to have a Mars Bar, cream cheese, or a glass of whole milk with your tablets.
- You also receive an injection once each week. This can occur in your doctor’s office, or you can give yourself the injection at home.

**Side Effects**

- You do not notice any side effects of either medication.

**HEALTH STATE H:**

Same hepatitis C and impact statements as Health State A.

**Treatment**

- In the hope of avoiding serious problems, you are being treated for hepatitis C. You are treated with oral and injectable medication.
- You take 18 tablets per day: 6 in the morning, 6 mid-day, 6 in the evening.
- You also receive an injection once each week. This can occur in your doctor’s office, or you can give yourself the injection at home.

**Side Effects**

- You do not notice any side effects of either medication.

**HEALTH STATE I:**

Same hepatitis C, impact, and treatment statements as Health State E.

**Side Effects: Anemia**

- As a side effect of your hepatitis C medication, you have anemia. Anemia means your blood cannot carry as much oxygen as it usually does.
- You sometimes feel tired and fatigued.
- You have shortness of breath when exerting yourself (for example, climbing stairs).
- These symptoms are often noticeable, but they are mild, and the anemia does not require treatment.

**HEALTH STATE J:**

Same hepatitis C, impact, and treatment statements as Health State E.

**Side Effects: Severe Anemia**

- As a side effect of your hepatitis C medication, you have severe anemia. Anemia means your blood cannot carry as much oxygen as it usually does.
- You often feel tired and fatigued.
- You have shortness of breath with regular activities (for example, walking).
- Your anemia symptoms require adjusting your treatment:
  - The dose of your hepatitis C medication is decreased. This makes your anemia symptoms less severe, but you still experience some fatigue and shortness of breath.
  - For example, you can still walk up stairs, but you feel very fatigued and short of breath afterwards.
  - Reducing the dose also reduces the effectiveness of your treatment.

**HEALTH STATE K:**

Same hepatitis C, impact, and treatment statements as Health State E.

**Side Effects: Flu-like Symptoms**

- As a side effect of your injectable medication, you have symptoms that feel like the flu for the first two days after every injection.
  - You have fever, headaches, muscle aches, joint pain, and shivers.
  - You feel fatigued. You feel hot and cold, as if you are freezing one moment and sweating the next.

**HEALTH STATE L:**

Same hepatitis C, impact, and treatment statements as Health State E.

**Side Effects: Rash**

- As a side effect of your hepatitis C medication, you have a dry, itchy, and scaly rash.
- Your rash appears on your arms and torso.
- The rash is uncomfortable, but it does not lead to more serious problems.
- This rash does not require prescription treatment.
- You can buy over-the-counter creams or lotions at a pharmacy to help make you feel a little more comfortable.

**HEALTH STATE M:**

Same hepatitis C, impact, and treatment statements as Health State E.

**Side Effects: Severe Rash**

- As a side effect of your hepatitis C medication, you often experience a red and itchy rash that sometimes has blisters.
- Your blisters feel hot or burning, and they leak fluid.
- This rash covers about half of your body.
- These symptoms require treatment. Your doctor prescribes a steroid cream to treat your rash.
- The cream causes your skin to feel oily and sticky.
- You must keep using this cream several times each day to keep your rash under control.
- Your rash never vanishes completely, but the cream helps make you feel a little more comfortable.

**HEALTH STATE N:**

Same hepatitis C, impact, and treatment statements as Health State E.

**Side Effects: Depression**

- As a side effect of your hepatitis C medication, you sometimes experience the following symptoms of depression.
  - You feel sad and hopeless.
  - You lose interest in your favorite activities.
  - You have difficulty sleeping.
  - You feel tired and have low energy.
  - You have difficulty thinking and concentrating.
